# The binding of negative emotional stimuli with spatial information in working memory: A possible role for the episodic buffer

**DOI:** 10.3389/fnins.2023.1112805

**Published:** 2023-03-22

**Authors:** Beatrice Cianfanelli, Antonino Esposito, Pietro Spataro, Alessandro Santirocchi, Vincenzo Cestari, Clelia Rossi-Arnaud, Marco Costanzi

**Affiliations:** ^1^Department of Human Sciences, LUMSA University, Rome, Italy; ^2^Department of Economy, Universitas Mercatorum, Rome, Italy; ^3^Department of Psychology, Sapienza University of Rome, Rome, Italy

**Keywords:** working memory, attention, object-relocation task, emotional valence, arousal

## Abstract

**Introduction:**

Remembering where negative events occur has undeniable adaptive value, however, how these memories are formed remains elusive. We investigated the role of working memory subcomponents in binding emotional and visuo-spatial information using an emotional version of the object relocation task (EORT).

**Methods:**

After displaying black rectangles simultaneously, emotional pictures (from the International Affective Pictures System) appeared sequentially over each rectangle. Participants repositioned the rectangles as accurately as possible after all stimuli had disappeared. During the EORT encoding phase, a verbal trail task was administered concurrently to selectively interfere with the central executive (CE). The immediate post-encoding administration of an object feature-report task was used to interfere with the episodic buffer (EB).

**Results:**

Only the EB-interfering task prevented the emotion-enhancing effect of negative pictures. The latter effect was not observed with a concurrent executive task.

**Discussion:**

Overall, our findings suggest that pre-attentive automatic processes are primarily involved in binding emotional and visuo-spatial information in the EB.

## 1. Introduction

Places in which negative experiences were encountered are usually well remembered. It is known that the emotional content of an experience facilitates consolidation processes, enhancing explicit long-term memories of that event (Richter-Levin and Akirav, 2003). However, the mechanism by which emotional stimuli are encoded and bound to spatial information in working memory remains unclear. We recently demonstrated that superimposing emotionally charged images on objects (black rectangles) that had to be relocated improved visuo-spatial memory for object position. Interestingly, this enhancing effect was only significant when both emotionally charged and neutral stimuli were presented during the same encoding trial. When all the presented images were unpleasant (negative valence), or when half of the images were pleasant (positive valence) and the other half were unpleasant, the effect of emotion vanished. These findings suggested that the emotional content of stimuli only affected memory for object position when neutral and emotional stimuli competed with one another (Costanzi et al., 2019).

Several studies support the competition hypothesis, suggesting that when information is initially processed, arousal prioritizes the processing of emotional stimuli through bottom–up perceptual salience and top–down relevance (Mather and Sutherland, 2011). Christianson (1992) hypothesized that negative stimuli processing occurs along a temporal continuum involving two stages: (i) a pre-attentive processing phase, assumed to be fast, unconscious, and independent of attentional resources, and (ii) a post-stimulus elaboration, a controlled process that occurs once attention has been directed to the emotional stimulus. Previous studies highlighted the central role of attention in processing emotional stimuli, by narrowing the focus of attention during encoding and facilitating their processing in memory systems (Nummenmaa et al., 2006; Riggs et al., 2011; Huntsinger, 2013; Kennedy et al., 2020). For example, Kensinger et al. (2007) found that when participants were presented with a complex visual scene that included a negatively arousing object placed on an otherwise non-emotional background, they remembered the negative arousing objects better than the neutral ones, and remembered the backgrounds presented with negative arousing objects worse than those presented with neutral ones. More recently, Kennedy et al. (2020) showed a performance impairment in a rotation detection task if an emotional picture appeared shortly before the rotated picture, confirming the existence of the typical “emotion-induced blindness” (Kennedy et al., 2020). Together these results suggest that the emotional content of visual stimuli can capture attentional resources when presented in both task-relevant and irrelevant conditions.

Despite this evidence, the role of attention in encoding emotional stimuli remains quite controversial. Several pieces of evidence show that memory recall for negative, but not for neutral and positive, pictures is unaffected by the administration of a concurrent attention-demanding task (divided attention condition), suggesting that the formation of negative valenced memories is largely dependent on automatic processes (Kern et al., 2005; Talmi et al., 2007; Migita et al., 2011). On the other hand, Pottage and Schaefer (2012) found that performance in a concurrent visual attention-demanding task mediated the enhancement of memory for affective information, suggesting that visual attentional processes play an important role in the encoding of emotional memories (Pottage and Schaefer, 2012). In a similar vein, Rossi-Arnaud et al. (2018) found a significant target-related advantage for all types of stimuli, regardless of their emotional valence, when investigating the attentional boost effect in the recognition of emotional pictures. Authors suggested that attention-dependent processes are involved in the encoding of emotional stimuli (Rossi-Arnaud et al., 2018). Finally, Kang et al. (2014) found that a divided attention condition impaired recognition of neutral and negative non-arousing words but not of negative-arousing ones, suggesting that negative valenced words can be encoded by either automatic or controlled processes, depending on the arousal level of the stimuli (Kang et al., 2014).

It is worth noting that the studies discussed so far have only considered memory for visual and verbal information, not for spatial information. To the best of our knowledge, only one study investigated the involvement of attentional mechanisms in processing emotional stimuli in visuo-spatial working memory. González-Garrido et al. (2015) combined an event-related potential (ERP) analysis with a behavioral study in which participants had to encode the position of neutral and emotional faces. They found that the amplitude of P2, an ERP component linked to attentional allocation, was higher for emotional than for neutral faces, and that emotional faces were better relocated than neutral ones. They suggested that visuo-spatial working memory relied on a domain-general attention-based mechanism, whereby the maintenance of spatial to-be-remembered locations might be influenced by the emotional content of the stimuli. It is important to note, however, that in the González-Garrido et al. (2015) study attentional control was not specifically manipulated and the emotional stimuli used in the behavioral task were only happy faces.

In the present study, we decided to investigate the role of different working memory subcomponents in binding negative stimuli, incidentally encoded, to the locations in which they were presented. According to Baddeley (2012) model of working memory, four main subcomponents have to be considered: the phonological loop (PL), responsible for rehearsal and temporary storage of verbal information; the visuo-spatial sketchpad (VSSP), which maintains visual and spatial information; the central executive (CE), a general-domain attentional module that controls ongoing processes; and the episodic buffer (EB), which binds information coming from different sources (Baddeley et al., 2012).

Here, we specifically target the roles of the CE and EB. The CE is a domain-general process that allows attentional resources to be allocated to the encoding of presented stimuli. Interfering with this process during encoding prevents exogenous information from entering the working memory system (Baddeley et al., 2012). The EB is a more recent addition to the working memory model and appears to be a later object-based storage process (Baddeley et al., 2011). Although tasks that assess its function are still being developed, there is increasing evidence that tasks requiring the binding of different features of stimuli (e.g., different visual features, like shape and color, color and position, different aspects of a geometric shape, and so on) require EB activity (see Nobre et al., 2013 for a review). Recently, Gao et al. (2017) found that administering a secondary feature reporting task during the working memory maintenance phase selectively disrupted binding while sparing memory for constituent features in a change detection task. These results suggested that EB is an independent storage buffer fueled by object-based attention (Gao et al., 2017). As concerns the role of EB in processing emotional information, although largely unexplored, it has been hypothesized that emotional valence may be rapidly detected and may act on information in the EB at both explicit and implicit levels (Baddeley, 2013). Mikels et al. (2008), for example, found that administering an affective interfering task, but not a cognitive one, impaired emotional working memory in an affective delayed-response task. The authors suggested that the EB may include a domain-specific component specialized in the active maintenance of emotional information.

We hypothesize that if a domain-general attention-based mechanism is involved in encoding the locations of negative-related objects, then a divided-attention condition should prevent the emotion-enhancing effect on spatial memory. To test this hypothesis, we used a dual task paradigm to interfere with the CE (verbal trail task; Fürst and Hitch, 2000) during the encoding phase of an emotional version of the object-relocation task (EORT; Costanzi et al., 2019). Negative pictures with high or low arousal levels and neutral pictures were used to investigate whether arousal modulates the switch between automatic and controlled processes in encoding emotional pictures. Moreover, the role of the EB was assessed by administering an object feature-report task (Gao et al., 2017)–known to interfere with the maintenance of bound visual information (e.g., colors and shapes) more than with the maintenance of single features–immediately after the EORT encoding phase. If the position of stimuli was bound with their emotional content in the EB, we would expect the object feature-report task to selectively interfere with the enhancing effect of emotional valence on memory for object location, while sparing memory for the single features (i.e., memory for both object locations and pictures’ identity).

## 2. Materials and methods

### 2.1. Participants, materials, and procedures

University students (197, of whom 152 females; age: 24.94 ± 3.77) voluntarily participated. All were Italian native speakers with normal or corrected-to-normal vision. The entire procedure was in accordance with the Helsinki Declaration and was approved by LUMSA University Ethical Committee.

In Experiment 1, EORT was run on a PC with a 17″ LCD monitor using a software programmed in Python. Because of COVID-19 pandemic restrictions, Experiments 2 and 3 were conducted online in a Google Meet virtual lab. Experimental tasks were run using jsPsych library (de Leeuw, 2015). In all experiments, the general procedure was alike to Costanzi et al. (2019). The EORT started with a 1000 ms fixation point, followed by a 1000 ms presentation of eight black rectangles in an array (165 × 128 px, 72 dpi). Four neutral and four negative pictures from the International Affective Picture System (IAPS; Lang et al., 2008) sequentially appeared superimposed, one at a time, on each rectangle (1000 ms; ISI: 250 ms). Participants were not informed about their occurrence. In Experiment 1, neutral and negative pictures differed in valence [neutral: 4.7 ± 0.25; negative: 3.1 ± 0.26; *t*_(6)_ = 8.85, *p* < 0.001] and arousal [neutral: 2.5 ± 0.17; negative: 5.7 ± 0.25; *t*_(6)_ = 12.34, *p* < 0.0001]. In Experiments 2 and 3, neutral and negative pictures differed in valence [neutral: 4.7 ± 0.32; negative: 3.5 ± 0.25; *t*_(6)_ = 5.57, *p* < 0.001] but not in arousal [neutral: 3.7 ± 0.18; negative: 3.8 ± 0.21; *t*_(6)_ = 0.41, *p* = 0.79].^1^ The images selected from the IAPS were simple photos depicting a single focal element and a stable background, with the exception of #9427 (Experiment 1) and #2590 (Experiments 2 and 3), which depicted negative complex scenes with multiple elements and an undefined background. Since neutral IAPS images are typically simpler in composition than negative ones (e.g., Marin et al., 2016), we also controlled for the visual complexity of the selected pictures. The following parameters were computed for each image and used as indexes of visual complexity: (i) the number of bytes and (ii) the number of blocks obtained with a quadratic tree decomposition (Gabrieli et al., 2022). This was computed with the pyaesthetics package,^2^ by setting 40 px as the minimum size and 10 as the minimum standard deviation for a block to be split. The statistical analyses of visual complexity (*t*-test) revealed no significant differences between neutral and negative pictures across all experiments—in Experiment 1: *t*_(6)_ = −0.16, *p* = 0.88 and *t*_(6)_ = 0.34, *p* = 0.74 for bytes and blocks number, respectively; in Experiments 2 and 3: *t*_(6)_ = 1.06, *p* = 0.33 and *t*_(6)_ = −0.48, *p* = 0.65 for bytes and blocks number, respectively.

**FIGURE 1:**
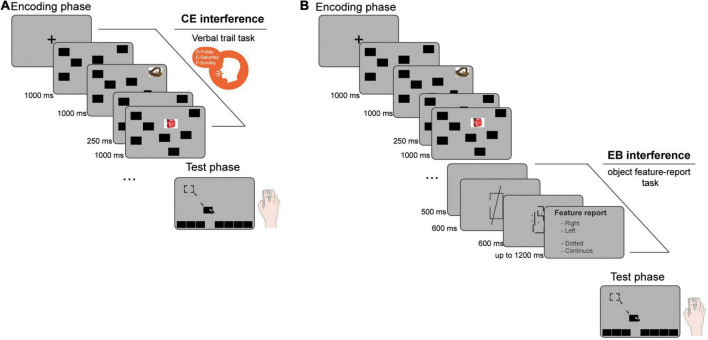
Schematic representations of the experimental procedures. **(A)** In Experiment 1 and 2 the concurrent administration of a verbal-trail task during the encoding phase of the emotional object relocation task (EORT) was used to interfere with the central executive (CE). **(B)** In Experiment 3, an object feature report-task immediately after the encoding phase of the EORT was used to interfere with the episodic buffer (EB).

In the test phase, all the black rectangles re-appeared at the bottom of the screen and participants had to relocate them as accurately as possible, using touchpad/mouse (Figure 1). Displacement error (dependent variable) was the distance in pixel between the center of the originally positioned object and the center of the closest relocated object.

Three hours after the test, participants were administered (i) an object-relocation task (ORT) which used pictures built by scrambling pixels of different colors in order to assess spatial working memory span, and (ii) a memory test for the incidentally learned pictures presented during the initial EORT. For the latter task, participants in Experiment 1 had to verbally describe the pictures (free recall). In Experiments 2 and 3, to facilitate online data collection, a two-alternative-forced-choice recognition task was used.

In Experiment 1, participants were randomly assigned to one of two experimental conditions (Figure 1A): (i) The CE-interference group (*n* = 29, 18 females) performed a concurrent verbal trail task: the experimenter provided a starting letter-day pair, and participants were required to continue the sequence with subsequent letter-day pairs (e.g., F-Sunday, G-Monday, etc.) until the encoding phase ended. (ii) The control group (*n* = 29, 18 females) performed only the EORT.

Experiment 2 (CE-interference group *n* = 30, 23 females; control group *n* = 26, 19 females) was similar to Experiment 1, with the exception that neutral and negative pictures were matched in terms of arousal.

In Experiment 3 participants were randomly assigned to one of two experimental conditions (Figure 1B): (i) The EB-interference group (*n* = 42, 39 females) performed an object feature-report task immediately after the EORT encoding phase. A blank screen (500 ms) was followed by a box-line stimulus formed with a square and an overlapping line oriented at 82° anti-clockwise to the horizontal plane. The entire stimulus was 360 × 396 px, lasted 600 ms and was placed in the center of the screen. The square presented a gap on either the right or the left vertical side, whereas the line could be solid or dashed. Following a mask of a random pattern of dots and lines (600 ms), participants had to report which side of the square presented the gap and how the line was. The EORT test phase began after an additional 500 ms of blank interval. (ii) The control group (*n* = 41, 34 females) watched the box-line stimulus but participants were explicitly instructed to ignore it.

### 2.2. Data analysis

In all experiments the displacement error was calculated as the distance (expressed in pixel) between the center of the originally positioned object and the center of the closest relocated object. The proportion of correctly recognized and/or recalled pictures was considered as an index for picture memory performance.

Two- or three-way ANOVAs and *t*-test were performed when appropriate on displacement error and on picture memory performance. Simple regressions analyses were also performed by considering picture memory performance as a predictor and displacement error as a criterion. All statistical analyses were performed with SPSS v.23 and GraphPad Prism (8.0.4) considering alpha = 0.05.

## 3. Results

**FIGURE 2:**
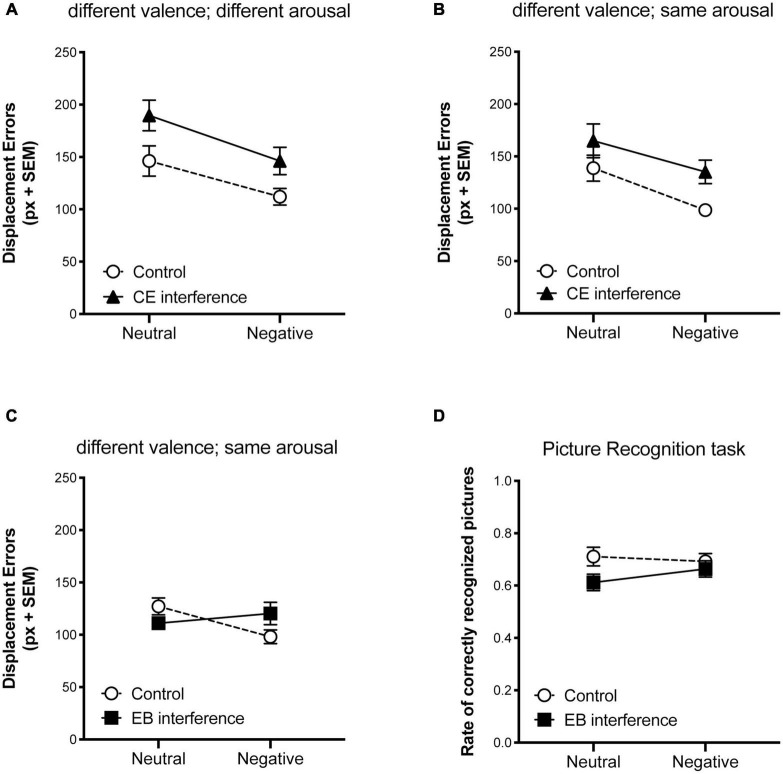
Effect of interfering tasks on visuo-spatial working memory performance in the EORT. Mean displacement errors obtained in controls and in and central executive (CE interference) suppression conditions **(A)** when negative pictures presented during the encoding phase of the EORT were more arousing than neutrals and **(B)** when negative and neutral pictures had similar levels of arousal. **(C)** Mean displacement errors obtained in controls and episodic buffer (EB interference) suppression condition. **(D)** Rate of correctly recognized pictures presented during the encoding phase of the EORT in controls and episodic buffer (EB interference) suppression condition. Vertical bars represent SEM.

In Experiments 1 and 2, two-way ANOVAs (2 × 2) were carried out on displacement errors, considering the experimental groups (CE-interference and control) as a between-subject factor and valence (negative and neutral) as a within-subject factor. In both experiments, significant main effects emerged for interference [Experiment 1: *F*_(1,56)_ = 9.079, *p* = 0.0039, $\eta_{p}^{2}$ 0.14; Experiment 2: *F*_(1,54)_ = 6.806, *p* = 0.0117, $\eta_{p}^{2}$ 0.11] and valence [Experiment 1: *F*_(1,56)_ = 9.219, *p* = 0.0036, $\eta_{p}^{2}$ 0.14; Experiment 2: *F*_(1,54)_ = 7.752, *p* = 0.0074, $\eta_{p}^{2}$ 0.13], while the interactions were not significant [Experiment 1: *F*_(1,56)_ = 0.1331, *p* = 0.7166, $\eta_{p}^{2}$ 0.002; Experiment 2: *F*_(1,54)_ = 0.1726, *p* = 0.6794, $\eta_{p}^{2}$ 0.003]. These results indicated that although CE interference reduced spatial working memory performance, perhaps increasing the cognitive load, emotional-related objects were better relocated than neutral-related ones in all experimental conditions (see Figures 2A, B). In both experiments, performance in ORT with scramble pictures did not differ among groups, indicating that the effect of interference on EORT performance cannot be ascribed to differences in spatial working memory span (*p* > 0.05; data not shown). Moreover, participants who underwent the CE interference showed high level of performance in the verbal trial task (96.8% ± 7.3 of correctness in Experiment 1 and 97.4% ± 6.7 in Experiment 2), indicating that the concurrent interfering task was effectively performed while encoding the EORT. Interfering with CE impaired memory for pictures in both experiments [Experiment 1: *F*_(1,56)_ = 12.76, *p* = 0.0007, $\eta_{p}^{2}$ 0.18; Experiment 2: *F*_(1,54)_ = 21.80, *p* < 0.0001, $\eta_{p}^{2}$ 0.3], whereas negative valence increased later retrieval only when arousal levels differed between negative and neutral pictures [Experiment 1: *F*_(1,56)_ = 20.93, *p* < 0.0001, $\eta_{p}^{2}$ 0.27; Experiment 2: *F*_(1,54)_ = 1.84, *p* = 0.18, $\eta_{p}^{2}$ 0.03].

In Experiment 3, two-way ANOVA (2 × 2) on displacement errors (Figure 2C) with groups (EB-interference and control) as a between subject factor and valence (negative and neutral) as a within subject factor, revealed a significant effect for the interaction [*F*_(1,81)_ = 5.93, *p* = 0.017, $\eta_{p}^{2}$ 0.07], but neither for group [*F*_(1,81)_ = 0.144, *p* = 0.7051, $\eta_{p}^{2}$ 0.002], nor for valence [*F*_(1,81)_ = 1.57, *p* = 0.21, $\eta_{p}^{2}$ 0.02]. *Post-hoc* analyses (Bonferroni’s multiple comparisons test) showed that negative-related objects were better relocated than neutral ones in the control group [*t*_(40)_ = 2.59, *p* = 0.01, $\eta_{p}^{2}$ 0.14], but not in the EB interference group [*t*_(41)_ = 0.84, *p* = 0.4, $\eta_{p}^{2}$ 0.017]. Also in this experiment, performances in ORT with scramble pictures did not differ between groups [t_(81)_ = 1.02; *P* = 0.31]. Moreover, participants who underwent the EB interference showed a high rate (82.4% ± 24.4.) of correct responses in the object feature-report task.

The two-way ANOVA carried out on recognition performance (Figure 2D) did not reveal any significant effects [group: *F*_(1,81)_ = 3.84, *p* = 0.053, $\eta_{p}^{2}$ 0.04; valence: *F*_(1,81)_ = 1.23, *p* = 0.27, $\eta_{p}^{2}$ 0.01; interaction: *F*_(1,81)_ = 0.27, *p* = 0.6, $\eta_{p}^{2}$ 0.003].

**FIGURE 3:**
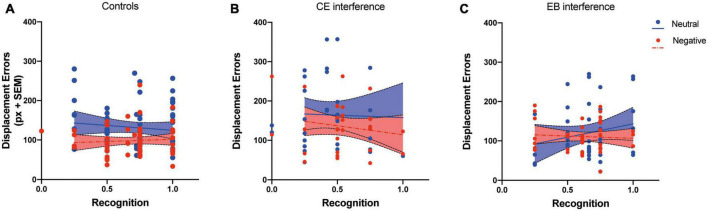
Relationship between the rate of correctly recognized pictures (Recognition) and the displacement errors in Experiments 2 and 3. Separate regression analyses for negative and neutral pictures were performed for controls **(A)**, CE-interference **(B)**, and EB-interference **(C)** groups.

A further regression analysis was performed by considering recognition performance as a predictor and displacement error as a criterion. We hypothesized that if attention-demanding processes modulate the binding of emotional pictures to spatial position, a significant correlation between memory for pictures and spatial working memory performance should be observed. Regression analyses performed on the results of Experiments 2 and 3 did not reveal any significant relation between the two variables in controls (*R*^2^ = 0.01, *p* = 0.41 for neutral picture condition and *R*^2^ = 0.004, *p* = 0.59 for negative picture condition), in the CE-interference group (*R*^2^ = 0.01, *p* = 0.86 for neutral picture condition and *R*^2^ = 0.003, *p* = 0.34 for negative picture condition), and EB-interference group (*R*^2^ = 0.004, *p* = 0.68 for neutral picture condition and *R*^2^ = 0.04, *p* = 0.21 for negative picture condition) (Figure 3). These results indicate that the relocation performance was unrelated to the explicit memory of pictures and suggest that the effect of negative pictures on spatial working memory is independent of attentional control.

To directly compare the effects of CE and EB interferences on single picture memory, we compared recognition accuracy between Experiment 2 and 3 participants. Three-way ANOVA (2 × 2 × 2) with valence (negative and neutral) as a within-subjects factor, and experiment (2 and 3) and group (control and interference) as between-subjects factors revealed significant main effects for both experiment [*F*_(1,135)_ = 7.0, *p* < 0.01, $\eta_{p}^{2}$ 0.05] and group [*F*_(1,135)_ = 26.98, *p* < 0.001, $\eta_{p}^{2}$ 0.17] as well as a significant interaction between the two factors [*F*_(1,135)_ = 8.65, *p* < 0.01, $\eta_{p}^{2}$ 0.06]. Bonferroni’s-corrected *t*-tests confirmed that interfering with the CE impaired memory for pictures compared to controls [*t*_(55)_ = −5.23, *p* < 0.001, $\eta_{p}^{2}$ 0.33], whereas interfering with the EB did not impact subsequent recognition performance compared to controls [*t*_(82)_ = 1.77, *p* = 0.08, $\eta_{p}^{2}$ 0.03]. Accordingly, recognition accuracy was lower in the CE-interference group than in EB-interference group [*t*_(71)_ = −4.05, *p* < 0.001, $\eta_{p}^{2}$ 0.19], whereas no difference emerged between the control groups of the two experiments [*t*_(66)_ = 0.02, *p* = 0.83, $\eta_{p}^{2}<$ 0.01].

Overall, this pattern of results indicates that interfering with the CE does not affect binding of negative pictures and spatial locations in working memory. Instead, interfering with EB activity selectively disrupted the binding. In contrast, when investigating memory for single pictures the opposite pattern emerged: interfering with the CE, but not with the EB, impaired later recognition performance.

Given the imbalance between the number of female and male participants in our experiments, gender was added as a covariate to all analyses reported above to test for the potential confounding effects of gender, following Schneider’s procedure for mixed designs experiments (Schneider et al., 2015). The results of the ANCOVA coincided in all experiments with those of ANOVA.

## 4. Discussion

The role of different working memory subcomponents in binding emotional and spatial information was investigated using an emotional version of the object relocation task (EORT). Results of Experiments 1 and 2 revealed that interfering with the CE during the encoding phase did not prevent the enhancing effect of incidentally presented negative pictures on object location memory. Although the CE interference impaired overall spatial working memory performance, negative-related objects were better relocated than neutral ones, regardless of the arousal level of negative pictures. These findings suggest that general attention-based mechanisms are not involved in binding emotional information to the location of objects.

On the other hand, the comparison of picture memory performance between Experiments 1 and 2 showed that the recollection of single images was impaired when domain-general attention resources were diverted from encoding. This pattern of results is in line with the known effect of divided attention during the encoding phase on subsequent recognition performance (e.g., Craik et al., 1996; Kang et al., 2014). Interestingly, in Experiment 1 negative high-arousing pictures were better remembered than neutral low-arousing one. No differences emerged in Experiment 2 where the arousal levels of both negative and neutral pictures were kept constant. This pattern of results is consistent with our previous findings that valence, rather than arousal, is involved in the prioritization of emotional stimuli in working memory access (Costanzi et al., 2019). It is also in line with theories proposing a primary role for arousal in the consolidation process of emotional memories (McGaugh, 2004; Mather and Sutherland, 2011).

In experiment 3 we found that interfering with the EB (i) completely and specifically prevented the emotion-enhancing effect exerted by negative pictures on spatial working memory performance, (ii) did not affect relocation performance of neutral-related objects–which was comparable to the control group, and (iii) did not affect recognition memory for both neutral and negative pictures presented during the EORT encoding phase. Moreover, the picture recognition accuracy of the EB-interference group (Experiment 3) was higher than the accuracy of the CE-interference group (Experiment 2). These results further support the idea that EB plays a key role in maintaining the bound representations of stimuli in working memory, whereas it does not influence memory for single features (Gao et al., 2017).

Findings from regression analyses revealed that spatial working memory performance of both control and interference groups did not correlate with memory for pictures. Although these analyses do not reveal any causal link between the two variables, the lack of a significant correlation suggests that the superior recall of the locations of negative pictures cannot be ascribed to explicit memory for those pictures.

Altogether, the findings of the present study suggest that automatic processes are primarily involved in modulating the emotion-enhancing effect of negative pictures on spatial working memory for object positions. For the present purposes, automatic processes can be considered as the opposite of domain-general attention-based processes, considering the latter as conscious mechanisms that drive the focus of attention. In this respect, interfering with attention-based processes means diverting conscious resources away from the main task (Schneider et al., 1982).

This interpretation appears to be consistent with several psychophysiological and behavioral studies showing that emotional stimuli are rapidly encoded through the activation of pre-attentive mechanisms that facilitate memory formation (Kern et al., 2005; Tamietto and De Gelder, 2010; Carretié, 2014; Arend et al., 2015; Kragel et al., 2021). From a psychophysiological standpoint, a main role for the subcortical regions (e.g., amygdala, pulvinar, basal ganglia, and superior colliculus) in prioritizing the selection of emotional stimuli in working memory has been proposed (Tamietto and De Gelder, 2010; Arend et al., 2015; Kragel et al., 2021). In particular, neuroimaging and lesion studies suggested that pulvinar is involved in both working memory and emotional information processing (Soto et al., 2007; Grecucci et al., 2010; Rotshtein et al., 2011; Kragel et al., 2021). Patients with lesions of the rostral part of pulvinar showed an impairment in spatial attention and visual filtering tasks, but not in emotional processing tasks, while patients with lesions of the medial part of pulvinar were impaired in emotional processing, but not in attention functions (Ward et al., 2002, 2005, 2007; Arend et al., 2008). Interestingly, neuroimaging studies reported a specific involvement of the medial pulvinar in working memory (Soto et al., 2007; Grecucci et al., 2010; Rotshtein et al., 2011). Based on these findings, a unique role for medial pulvinar in binding emotionally relevant stimuli with information held in working memory has been envisaged (Arend et al., 2015). More recently Kragel et al. (2021) found a selective role for the collicular-pulvinar-amygdala pathway in mediating the unconscious affective responses to visual stimuli (Kragel et al., 2021). These findings add new evidence to behavioral studies, indicating that the processing of emotional information is often prioritized and independent of attentional resources (Carretié et al., 2004; Vuilleumier, 2005; Tamietto and De Gelder, 2010).

From a behavioral point of view, Phillips et al. (2008) found that performance accuracy in a two-choice discrimination task requiring participants to decide whether two emotions were the same or different was relatively unaffected by the administration of a concurrent task taxing working memory capacity, supporting the hypothesis that the encoding of emotional stimuli is automatic and does not require attentional resources (Phillips et al., 2008; Tsouli et al., 2017). In discussing how the emotional valence of stimuli might be processed in working memory tasks, several pieces of evidence suggested the existence of dedicated subcomponent. Mikels et al. (2008) found that an emotional, but not a cognitive, interfering task impaired emotional working memory performance, suggesting that working memory may include a domain-specific component specialized in the processing of emotional information. Along the same lines, Baddeley et al. (2012) proposed the existence of a dedicated “hedonic detector,” which automatically assesses the emotional values of incoming sensory stimuli. This process modulates how information is selected, represented, and stored in working memory (Baddeley, 2007; Baddeley et al., 2012; Ribeiro et al., 2018). In his seminal work, Baddeley (2007) hypothesized that the hedonic detector evaluates the valence of incoming stimuli by setting a neutral point based on one’s mood. As a result, inducing a negative mood leads to a more negative assessment of stimuli that must be processed in working memory (Baddeley et al., 2012). Ribeiro et al. (2018) extended Baddeley’s hypothesis by pointing out that the “hedonic detector” would explain the impact that emotional stimuli processing might have on working memory performance. Our results support the idea that the hedonic detector can automatically reveal the valence of the emotional stimuli processed by working memory. We also envisage the hedonic detector being integrated in the working memory model through a direct connection with the EB.

General attention-demanding processes can certainly modulate the enhancing effect of emotional pictures on working memory, especially when participants are instructed to pay attention to emotional stimuli relevant for solving the working memory task (Kensinger and Corkin, 2003; Schupp et al., 2007; Ziaei et al., 2014). After all, the EB can be also fueled by the CE through attention-based mechanisms (Baddeley et al., 2010; Gao et al., 2017). However, we believe that the valence of emotional stimuli—revealed by the hedonic detector—may automatically tag the location of objects retained in the EB, leading to a better memory for that location. This interpretation is also consistent with findings showing that conditions requiring binding are less sensitive to attention-demanding concurrent tasks than conditions requiring retention of single features, such as color or shape (Baddeley et al., 2010).

Alternatively, since in our experiments we interfere with CE by administering an attention-demanding task during the encoding phase of EORT, attentional control may be required in binding emotional and spatial information in a later stage of information processing (i.e., during the maintenance or retrieval phases). However, previous results have shown that interfering with attentional mechanisms after the encoding phase of visuo-spatial working memory tasks has little impact on feature binding memory (Johnson et al., 2008; Delvenne et al., 2010; Zhang et al., 2012). Another caveat when interpreting our findings is the different domains of the interfering tasks: verbal for CE and visual for EB. It is possible that the different modalities in which the interfering tasks were administered influenced our results. However, because the CE-mediated attention that we interfered with in Experiments 1 and 2 is assumed to be domain-general (Allen et al., 2006; Gao et al., 2017), we would expect similar results when presenting a secondary task with a different modality. Indeed, there is evidence showing that the concurrent administration of two attention-demanding tasks (dual-task condition) taxing different sensory modalities (i.e., visual and verbal) lead to an interference because the two tasks depend on the activation of the same brain areas (involved in domain-general attention processing: Klingberg, 1998; Loose et al., 2003). Regarding EB, administering either auditory or visual secondary tasks in the maintenance phase of a visual working memory task impaired memory for binding (Zokaei et al., 2014). In addition, the administration of a visual object feature-reporting task affected the binding of verbal to visual information (Gao et al., 2017). These results suggest that EB activity in maintaining bound representations may be inhibited regardless of the modality (i.e., verbal or visual) with which the secondary task is presented. We therefore conclude that differences in the modality (i.e., verbal vs. visual) and/or in the phase (encoding vs. maintenance) in which the interfering tasks were administered may have had negligible effects on the results obtained in the present study. However, future experiments would be necessary to better assess the effect of domain- and phase-specific interference in modulating the binding of spatial and emotional information.

Richter-Levin and Akirav (2003) suggested that emotional stimuli may trigger subcortical neuromodulatory systems, which in turn can modulate (tag) the synaptic activity of neurons in other brain regions. This “emotional tagging” would affect memory formation by influencing the sorting of important stimuli among less important ones (Richter-Levin and Akirav, 2003). Coherently with the “emotional tagging” hypothesis, we propose that the emotional content of stimuli operates in the early phase of information processing, allowing emotional-related stimuli to be prioritized in accessing the working memory system, through the connection between the hedonic detector and EB. As a result, memory for the location of the emotional stimulus improves. This interpretation of our findings may provide a neurobiological and cognitive explanation for affective working memory, though further research is needed to confirm this hypothesis.

## Data availability statement

The raw data supporting the conclusions of this article will be made available by the authors, without undue reservation.

## Ethics statement

The studies involving human participants were reviewed and approved by CERS, LUMSA University. Date of approval: December 10, 2019. The patients/participants provided their written informed consent to participate in this study.

## Author contributions

BC and MC developed the idea for this study and drafted the manuscript. BC, AE, PS, AS, VC, CR-A, and MC contributed conception and designed the study. BC and AE collected the data and organized the database. BC, AE, CR-A, VC, and MC analyzed and interpreted data. PS and AS contributed to the discussion of content-related issues and to the critical revision of the article and wrote sections of the manuscript. MC and CR-A wrote the final version of the manuscript. All authors contributed to the manuscript revision, read, and approved the submitted version.

## Footnotes

1 IAPS pictures were selected by considering valence (from unpleasant to pleasant) and arousal (from calm to exciting) evaluated on a 9-point scale. In study 1, pictures were:

- Neutral: *#7035* (4.98; 2.66), *#7060* (4.43; 2.55), *#7110* (4.55; 2.27), *#7491* (4.82; 2.39).
- Negative: *#1220* (3.47; 5.57), *#6244* (3.09; 5.68), *#9427* (2.89; 5.5), *#9630* (2.96; 6.06).

In studies 2 and 3, pictures were:

- Neutral: *#2214* (5.01; 3.46), *#2484* (5; 3.75), *#7011* (4.52; 3.81), *#7290* (4.37; 3.87).
- Negative: *#2590* (3.26; 3.93), *#2722* (3.47; 3.52), *#7078* (3.79; 3.69), *#9110* (3.76; 3.98).

Valence and arousal values are reported for each picture in parentheses.

2
https://github.com/Gabrock94/pyaesthetics
